
JOURNAL INFORMATION
==============================
NLM Title Abbreviation: Atlanta Med Surg J (1884)
NLM Journal ID: 101251623

Atlanta Medical and Surgical Journal (1884)

Publisher: Biomedical Journal Digitization Project

ARTICLE INFORMATION
==============================
PMCID: PMC9199290
PMID: 35829589
Article version: 1
Subjects: Selections and Abstracts

Selections and Abstracts

Print publication date: 1898 Dec
Volume: 15
Volume Series: New Ser
Issue: 10
First page: 706
Last page: 720
License: This work is free of known copyright restrictions. For more information, please see PMC Back Issue Digitization.
License URL: https://creativecommons.org/publicdomain/mark/1.0/

==============================

### SELECTIONS AND ABSTRACTS.

---

### The Preservation of the Hymen.

Howard A. Kelly, M.D. (*American Journal of Obstetrics*): In Celsus's "De Medicina," written about the time of Christ, in the chapter on calculi, after declaring that small calculi may escape spontaneously through the shorter and more relaxed urethra, he says that in most cases it will be necessary to apply the same methods of hooking the calculus down and holding it against the neck of the bladder while incising, as in men, but he adds the caution that in the virgin the finger must be introduced per anum, as in the male, while in married women it may be introduced into the vagina.

Severin Pineau, in his work written at the end of the sixteenth century on the signs of virginity, quotes the ancients as his authority for the dictum, "it is criminal to rupture the hymen" ("magnum est crimen perrumpere virginis hymen").

And such has been the attitude of the profession through all the centuries of the past; honorable men have carefully guarded as sacred the rights and the interests of the young women who have trusted their persons to their professional care, and the barrier which nature has erected at the vaginal introitus as the sole *prima facie* evidence of virginity has been preserved intact.

I wish now to urge, in the first place, that it has remained for our day and generation, at a time when the study of the diseases of women has become a fashion, for practitioners of medicine to ruthlessly disregard all moral considerations and make digital examinations of young women with pelvic pains at the menstrual period, or those presenting any abnormality in the menstrual flow or complaining of a leucorrhea.

I know not how else to characterize this *cacoethes examinandi*, this reckless habit of investigating the sexual organs of young women, than as a species of rape.

I know, for example, of a large institution in our city entrusted with the education of young women from all parts of the country. In numerous instances young girls with purely functional dysmenorrhea are taken to a physician, who examines them, inserts specula, and institutes treatments where, as I have had occasion to verify, there is actually no discoverable local disorder whatever.

Other young women are constantly coming to me from a distance with the statement that they have "ovarian disease," or "tubal disease," or "anteflexion of the womb," who are perfectly sound, and who ought never to have been examined at all; and I have thus to record in my case-book in numerous instances "no pelvic disease" or "metrophobia"—a word I have coined to designate those who are fully assured they have some grave "womb trouble," but are, notwithstanding, perfectly sound.

Aside from all the hospital cases I have seen, I find in my private case-book the records of twenty-eight cases with no pelvic disease out of a total of 550 in all, about five per cent.

While male physicians are great offenders in this respect, many of the women who practice medicine are far worse; indeed, they often seem to possess no conscience whatever in dealing with these sacred interests of their own sex.

A young woman with a natural sense of shame, but utterly guileless of any knowledge of anatomy, feels that it is easier for her to speak of her pelvic discomforts to one of her own sex; but the woman doctor (with many noble and conspicuous exceptions, I am happy to say) then feels, on her part, that it is necessary, in order to make a complete investigation of every case, to inspect the genitals and to insert her finger into the vagina; she generally ends by putting in a speculum, too, and tampons, and so begins a never-ending course of so-called "treatments," during which the natural ups and downs of health encourage the trusting victim to think at first that the "treatments" are benefiting her, and then, when the pains recur, that there surely is something at fault, and so it continues for years. Sooner or later, in many cases, an infection is introduced, and from having no disease at all she is inoculated with a salpingitis, and she is fortunate, finally, if she escapes a radical operation removing her uterine tubes and her ovaries. The records of this country within the past twenty years would show, I verily believe, thousands of such victims, at first unnecessarily insulted, and then robbed of their distinctive organs of sex for imaginary ailments or diseases acquired.

Such is the character of this evil; of its extent no one can do more than roughly estimate. My own experience leads me to conclude that these vicious practices are both general throughout our country, and that they affect our young womanhood to the extent of inflicting an unnecessary injury upon many thousands yearly.

In case an examination is actually necessary, it must be made by one who is thoroughly competent to decide at once whether or not there is any disease present, and who will be able to proceed at once to do all that may be required to relieve it. By dilating a cervix, or rupturing a large Graafian-follicle cyst, or breaking up adhesions, oftentimes all can be done at once which it is possible to do at all, and the patient will be spared the distress of useless and endless so-called "treatments," which consist in the application of drugs to the vaginal walls or to the cervix, anatomical structures distant from and quite distinct from the supposedly affected organs.

I would urge that there are satisfactory methods of examining young women which need shock no sensibilities and which inflict no injury upon any organ.

In the first place, if the patient has not been examined and treated before, and an examination is necessary, it is my invariable practice to propose to do this *under complete anesthesia,* in this way securing a perfect relaxation, with every facility for making the minutest investigation which it is possible to make, short of inspection through an abdominal incision. The anesthetic obviates the inevitable resistance of the abdominal walls, and the examination leaves behind it no disagreeable memories.

In the second place, when the patient is anesthetized *the examination must be conducted through the rectum.* The cervix can be easily palpated through the recto-vaginal wall; and as for the body of the uterus, the ovaries, and the tubes, they cannot be clearly palpated in any other way. The rectal examination is therefore not only to be recommended because it spares the hymen, but because *it is actually indispensable to a thorough investigation.*

I have shown on another occasion that the most minute examination may be made by the rectum if the pelvic organs are first skeletonized by putting the patient in the knee-breast position to let in the air and so getting rid of the intestines. After doing this the examination is conducted in the dorsal position. If the finger is not more than 6.5 centimeters in circumference, it may often be inserted into the vagina, slowly and with extreme care, without rupturing the hymen; this is, however, not a good rule, for the tactile sense of many men seems to be so blunt that there is no appreciation of resistance when delicate structures are being investigated, and harm is done unwares.

In the third place, if it is necessary to dilate and to curette the uterus, this may be done by introducing a finger into the rectum and locating the cervix, and then introducing a pair of tenaculum forceps into the vagina and carefully opening them, and catching the cervix and drawing it down to the outlet, where it may then be dilated and curetted, in many cases, without injuring the hymen.

---

### SOME OBSERVATIONS ON BRAIN ANATOMY AND BRAIN TUMORS.

Dr. William C. Krauss, of Buffalo, read a paper at the ninety-second annual meeting of the Medical Society of the State of New York, Albany, January 25, 1898, with the above title.

He called attention (1) to the difficulty in remembering the gross anatomy of the brain, and (2) to the almost universal presence of optic neuritis in cases of brain tumor.

He attempted to overcome the difficulty in regard to the anatomy of the brain by formulating the following rules, which are somewhat unique and original, and at the same time easily remembered:

*Rule of Two.*—1. The nerve centers are divided into two great divisions, (1) encephalon, (2) myelon. 2. The encephalon is divided into two subdivisions , (1) cerebrum, (2) cerebellum. 3. The cerebrum, cerebellum, and myelon are divided into two hemispheres each, (1) right, (2) left. 4. The encephalon is indented by two great fissures, (1) longitudinal, (2) transverse. 5. Into these two great fissures there dip two folds of the dura, (1) falx cerebri, (2) tentorium cerebelli. 6. There are two varieties of brain matter, (1) white, (2) gray.

*Rule of Three.*—1. There are three layers of membranes surrounding the brain, (1) dura, (2) arachnoid, (3) pia. 2. Each hemisphere is indented by three major fissures, (1) sylvian, (2) rolandic or central, (3) parieto-occipital. 3. Three lobes, frontal, temporal and occipital, on their convex surface are divided into three convolutions each—superior, middle and inferior, or first, second and third. 4. There are three pairs of basal ganglia, (1) striata, (2) thalami, (3) quadrigemina. 5. The hemispheres of the brain are connected by three commissures, (1) anterior, (2) medi, (3) post-commissure. 6. The cerebellum consists of three portions, (1) right, (2) left hemisphere, (3) vermes. 7. There are three pairs of cerebellar peduncles, (1) superior, (2) middle, (3) inferior. 8. The number of pairs of cranial nerves, in the classifications of Willis and Sommering, can be determined by adding three to the number of letters in each name; that of Willis making nine, and that of Sommering making twelve, or the name containing the more letters has the larger number of pairs of nerves, and *vice versa.* 9. The cortex of the cerebellum is divided into three layers of cells, (1) granular, (2) Purkinje's cells, (3) a molecular layer.

*Rule of Five.*—1. Each hemisphere is divided externally into five lobes, of which four are visible, (1) frontal, (2) parietal, (3) temporal, (4) occipital; and one invisible, (5) insula (isle of Reil). Roughly speaking, the visible lobes correspond to the bones of the cranium; that is, the frontal lobe is underneath the frontal bone, the parietal lobe beneath the parietal bone, etc. 2. The brain contains five ventricles, of which four are visible—the right and left, or first and second, the third and fourth; and one invisible, the fifth, or pseudo-ventricle. 3. The cortex of the brain contains five distinct layers of ganglion cells.

Studying carefully one hundred cases of brain tumor in which an ophthalmoscopic examination had been made for the presence or absence of choked disc (optic neuritis), Dr. Krauss announced the following conclusions:

1. Optic neuritis is present in about 90 per cent. of all cases of brain tumor.
2. It is more often present in cerebral than in cerebellar cases.
3. The location of the tumor exerts little influence over the appearance of the papillitis.

4. The size and nature of the tumor exerts but little influence over the production of the papillitis.

5. Tumors of slow growth are less inclined to be accompanied with optic neuritis than those of rapid growth.

6. It is probable that unilateral choked disc is indicative of disease in the hemisphere corresponding to the eye involved.

7. It is doubtful whether increased intracranial pressure is solely and alone responsible for the production of an optic neuritis in cases of brain tumor.—*Philadelphia Med. Journal.*

---

(*From the Medical Review, St. Louis, Mo., February* 26, 1898.)

### OBSERVATIONS UPON THE TREATMENT OF SOME CASES OF NEURASTHENIA.*

BY JEROME K. BAUDUY, M.D., LL.D., ST. LOUIS, MO.
Professor Nervous and Mental Diseases, and of Medical Jurisprudence, Missouri Medical College.

CLINICAL REPORT
BY KEATING BAUDUY, M.D.,
ST. LOUIS MO.

MICROSCOPIC REPORT
BY C. FISCH, M.D.,
ST. LOUIS, MO.

That chalybeates, more especially the *organic* salts of iron, constitute an essential indication in the successful treatment of some cases of neurasthenia, especially in the female, where functional menstrual derangements exist, is to my mind *an indisputable fact.* They produce conditions, oftentimes not attainable by the inorganic preparations for many reasons, which experience and reflection clearly demonstrate.

In a recent clinical study of this affection, my conclusion, as above stated, is fully justified and corroborated by the microscopical blood examinations conducted by my esteemed and skillful friend Dr. C. Fisch. That cerebro-spinal anemia is a frequent important concomitant, if not an essential etiological factor of neurasthenia, I hardly think admits of cavil.

The clinical histories of appended cases were compiled by my son, Dr. Keating Bauduy, chief of the Neurological Clinic at St. John's Hospital, under whose direct supervision the investigations were conducted. That the ratio, or number of red blood corpuscles, and the percentage of hemoglobin were deficient in the normal standard of these cases, prior to the treatment, *is incontestable,* as shown by the microscope. That several of these cases to be enumerated showed marked improvement, even after one or two weeks' treatment, is moreover revealed in the same manner, and which for rapidity of effect is quite an exceptional, if not a startling therapeutic result, when compared with some of the prior and more established methods of treatment. That many of these cases presented unmistakable evidence of satisfactory improvement, from both a subjective and objective standpoint, was quite as notable as the permanent character of their general amelioration. That the ordinary tonics had in some instances been administered with neugatory results, while pursued along the old lines of authoritative medication, seems quite manifest.

My only explanation of the *surprising results* in the cases herein cited, where the usual officinal class of remedies had formerly been ineffectually essayed, was the superinduction as is so frequently the case of disturbed digestion and assimilation; results but too familiar and disappointing to professional experience. Aside from the disturbances just mentioned, the development of headache, constipation, etc., frequently obviates their further administration.

When, a few years ago, my attention was called to Gude's preparation of *"Liquor Mangano-Ferri Peptonatus, Gude,"* (Pepto-Mangan) so extensively used and highly extolled in Germany, with my usual antipathy for new remedies, I reluctantly gave it a trial, anticipating that I would necessarily have to combat the usual disappointing effects of most of the other preparations of iron. The results, however, were *indeed a surprise to myself*, for the concomitant deranging sequelæ were so slight, that but in very few instances in *my extensive* utilization and experience with this special pharmaceutical preparation was I obliged to discontinue it. My experience having led me to believe that iron and manganese in combination are both indicated in the vast majority of cases of neurasthenia, this particular remedy, *I am convinced, will prove a great boon both to the patient and the physician.* While it is maintained by some that the hemoglobin of the red blood corpuscle manganese is present, as well as iron, I have for many years procured results with a combination of both, not directly obtainable with one alone. We know, however, that manganese gives off oxygen to a greater degree than iron, and it has been argued that for this reason its internal exhibition might correspondingly increase assimilation.

Dr. Fisch's appended microscopical report shows that the increase in the percentage of hemoglobin, in many of this series of cases, is far in excess of the proportionate increase of the red blood corpuscles. *This fact I deem of greater importance as to the effectiveness of the medicine*, because the count cf the blood corpuscles is to a certain extent relative, and the size varies greatly in different cases, and for other reasons the same amount of blood plasma contains different numbers of red cells; hence I would particularly lay stress upon the proportionate increase of the hemoglobin as the more important factor. The *notable* and *astonishing improvement* of these cases, when placed upon this preparation, led me to their closer scrutiny, as well as microscopic observation. Before concluding, I *wish particularly* to call attention to the fact of the absence of digestive disturbances and necessary consequent interference in the assimilation. All other unpleasant complicating results were notable by their absence. Of course we do not consider the remedy applicable to cases of lithemic neurasthenia, nor in any manner a *specific* in any variety of neurasthenia. In many cases the addition of arsenic and strychnia greatly increases the efficacy of the preparation. I must also take cognizance of the salient fact of the rapidity with which a large number of female neurasthenics, under our treatment, who have suffered with marked functional menstrual derangements, have attained a normal condition under the administration *of this most elegant combination of iron and manganese.*

As it would be tedious and monotonous to present an exhaustive citation of a multiplicity of clinical cases, I have confined myself to a recital of a few typical ones:

CLINICAL REPORT

BY KEATING BAUDUY, M.D.

CASE 1.—Mrs. S., aged 32 years, mother of three children, came to me in a pitiable mental condition, and had in her arms a nursing hydrocephalic child, five months old. Her mental depression approached a type of veritable melancholia. My first idea was to advise that the child be weaned, and then place her upon the classical opium treatment for melancholia. This was her third child, and like all mothers, she clung to the life of her unfortunate with characteristic tenderness. Therefore she bluntly insisted upon my candid opinion, as to whether the weaning of the baby might prove fatal. Knowing, as I did, that the life of the child was simply a question of a period of short duration in either case, I so informed her; nevertheless, I insisted that the best hope for her recovery was to wean it. This she refused to do, and after Dr. Fisch had made a blood examination and pronounced her highly anemic, I reluctantly undertook the case. Aside from her mental depression, physical lassitude, and marked pallor, the "casque neurasthenique" symptom was a dominant feature in her case. Any effort to perform her usual household duties produced sensations of cerebral fullness, and persistent pain in the vertex. She even confessed that the idea of suicide had of late frequently haunted her. Under the administration of "Pepto-Mangan," with no other treatment, after the short period of fifty-two days, she was discharged fully restored to her normal condition. Microscopic report showed a relative gain in number of red blood corpuscles of 34 per cent.; hemoglobin, 44.5 per cent.

CASE I.

| | | |
|---|---|---|
| I. Examination. (Beginning of Treatment.) Date, November 17, 1897. | Red Corpuscles | 3120000 |
| | Hemoglobin, per cent. | 52 |
| II. Examination. Date, December 2, 1897. | Red Corpuscles | 3400000 |
| | Hemoglobin, per cent. | 54 |
| III. Examination. Date, December 26, 1897. | Red Corpuscles | 4130000 |
| | Hemoglobin, per cent. | 67 |
| IV. Examination. Date, January 8, 1898 | Red Corpuscles | 4245000 |
| | Hemoglobin, per cent. | 75 |
| Duration of Treatment | | 52 days |
| Gain (Absolute) | Red Corpuscles (in 1000's) | 1125 |
| | Hemoglobin, per ct. | 23 |
| Gain (Relative) | Red Corpuscles, per cent | 34 |
| | Hemoglobin, per ct. | 44.5 |

CASE 2.—Mrs. Sim, aged 23 years, mother of two children, youngest six months and nursing. About the fourth month of her last pregnancy she was troubled with dyspnea. Gave history of instrumental delivery, followed by puerperal eclampsia. Great loss of blood during birth of child. Two months later, abscesses developed in each breast, and patient was confined to bed during a period of ten weeks. Case presented typical manifestations of neurasthenia, also characteristic apprehensions, with preternatural emotional mobility. Constant cephalalgia in vertical region, persistent parasthesia in extremities, mouth and tongue, were also present. She was intensely pale with every appearance of profound anemia. Aside from a mild laxative which was given to obviate constipation—an obstinate feature in her case—nothing was administered, save "Pepto-Mangan." After a period of treatment of forty-nine days I discharged her, as she evinced none of the symptoms which formerly existed. A notable feature was the corresponding improvement of the child, notwithstanding the fact that I had previously insisted upon its being weaned, which she had, nevertheless contrary to my instructions, continued to nurse. Microscopic report showed a relative gain: red blood corpuscles, 19 per cent.; hemoglobin, 27 per cent.

CASE II.

I. EXAMINATION.
(Beginning of Treatment.)
Date, November 20, 1897.
Red Corpuscles........3470000
Hemoglobin, per cent.. 60

II. EXAMINATION.
Date, December 22, 1897.
Red Corpuscles.... . 3980000
Hemoglobin, per cent.. 69

III. EXAMINATION.
Date, January 8, 1898.
Red Corpuscles.... ...4120000
Hemoglobin, per cent.. 76

DURATION OF TREATMENT......49 days

| | | |
|---|---|---|
| GAIN (Absolute) | Red Corpuscles (in 1000's)............. | 650 |
| | Hemoglobin, per ct. | 16 |
| GAIN (Relative) | Red Corpuscles, per cent................ | 19 |
| | Hemoglobin, per ct.. | 27 |

CASE III.

| | | |
|---|---|---|
| I. EXAMINATION. (Beginning of Treatment.) Date, November 26, 1897. | Red Corpuscles | 3720000 |
| | Hemoglobin, per cent.. | 61 |
| II. EXAMINATION. Date, January 22, 1898. | Red Corpuscles | 4135000 |
| | Hemoglobin, per cent.. | 70 |
| DURATION OF TREATMENT | | 57 days |
| GAIN (Absolute) | Red Corpuscles (in 1000's) | 415 |
| | Hemoglobin, per ct. | 9 |
| GAIN (Relative) | Red Corpuscles, per cent | 11 |
| | Hemoglobin, per ct. | 15 |

CASE IV.

| | | |
|---|---|---|
| I. EXAMINATION. (Beginning of Treatment.) Date, November 26, 1897. | Red Corpuscles | 2807000 |
| | Hemoglobin, per cent.. | 46 |
| II. EXAMINATION. Date, December 17, 1897. | Red Corpuscles | 3200000 |
| | Hemoglobin, per cent.. | 50 |
| III. EXAMINATION. Date, January 4, 1898. | Red Corpuscles | 3250000 |
| | Hemoglobin, per cent.. | 56 |
| IV. EXAMINATION. Date, January 8, 1898. | Red Corpuscles | 3875000 |
| | Hemoglobin, per cent.. | 68 |
| DURATION OF TREATMENT | | 41 days |
| GAIN (Absolute) | Red Corpuscles (in 1000's) | 1068 |
| | Hemoglobin, per ct.. | 22 |
| GAIN (Relative) | Red Corpuscles, per cent | 38 |
| | Hemoglobin, per ct. | 47 |

CASE 3.—D. G., aged 25 years, unmarried. Suffered from nervous headache for past year. Vaso-motor disturbances evidenced by alternate flushings and pallors, heat and cold. Atonic dyspepsia. Irregularity of bowels. Disturbed sleep. Depressed physical condition, corresponding weak pulse. After taking "Pepto-Mangan" fifty-seven days, reported feeling generally improved. Digestion was better, pulse stronger and headaches greatly diminished in intensity. Vaso-motor disturbances disappeared. Microscopic examination showed a relative gain: red blood corpuscles, 11 per cent.; hemoglobin, 15 per cent.

CASE 4.—Miss S., aged 28 years, presenting many of the well-defined symptoms of neurasthenia, was in a condition of profound mental and physical weakness. The history showed that since our great cyclone of May 27, 1896, she had never been her normal self, and was unable to perform any sustained mental or physical strain. Dating from that episode she had always worried, and was constantly the victim of peculiar forebodings. Insomnia and general malaise were cardinal symptoms. My diagnosis was what has been termed "cyclone neurosis," of which I have seen numerous cases. Menorrhagia existed to an alarming extent for which I accordingly recommended rest and the recumbent posture during her periods. Because of the pronounced insomnia, I prescribed a nightly dose of hyoscyamine and sulfonal during the first week of treatment as a hypnotic, which constituted the only medication other than "Pepto-Mangan." After having taken the latter for forty-one days, I discharged her from treatment, as she had passed her last menstrual period after a normal flow of three days, her pallor having given way to rosy cheeks and her physical and mental condition being entirely satisfactory. Microscopic report showed a relative gain: red blood corpuscles, 38 per cent.; hemoglobin, 47 per cent.

CASE 5.—Mr. C., aged 21 years, unmarried. Highly anemic, very pale. Anorexia and insomnia persistent. Physical condition greatly depressed. Cardinal feature was a sexual hypochondrical tendency. Gave history of excesses both alcoholic and sexual. Aside from advice as to the necessity of leading a moral life, and abstaining from all stimulants, gave no medicine but "Pepto-Mangan," with the addition of arsenic and strychnia. After fifty-seven days of treatment, patient was much benefited. Microscopic report showed a relative gain: red blood corpuscles, 9 per cent.; hemoglobin, 27 per cent.

CASE 6.—Mrs. D., aged 36 years, married; five children. Since birth of last child, eighteen months ago, has been in state of profound nervous prostration. Previously resisted ordinary tonic and constructive treatment. Menorrhagia was the dominant feature of the case. After taking "Pepto-Mangan" for fifty-one days patient evinced more improvement than during any stated time throughout the past eighteen months. Last menstruation approached the normal

CASE V.

| | |
|---|---|
| I. EXAMINATION. (Beginning of Treatment.) Date, November 26, 1897. | |
| Red Corpuscles | 3670000 |
| Hemoglobin, per cent. | 44 |
| II. EXAMINATION. Date, December 14, 1897. | |
| Red Corpuscles | 3700000 |
| Hemoglobin, per cent. | 42 |
| III. EXAMINATION. Date, January 8, 1898. | |
| Red Corpuscles | 3990000 |
| Hemoglobin, per cent. | 54 |
| IV. EXAMINATION. Date, January 22, 1898 | |
| Red Corpuscles | 4010000 |
| Hemoglobin, per cent. | 56 |
| DURATION OF TREATMENT. | 57 days |
| GAIN (Absolute) — Red Corpuscles (in 1000's) | 310 |
| GAIN (Absolute) — Hemoglobin, per ct. | 12 |
| GAIN (Relative) — Red Corpuscles, per cent. | 9 |
| GAIN (Relative) — Hemoglobin, per ct. | 27 |

CASE VI.

| | |
|---|---|
| I. EXAMINATION. (Beginning of Treatment.) Date, November 26, 1897. | |
| Red Corpuscles | 3450000 |
| Hemoglobin, per cent. | 60 |
| II. EXAMINATION. Date, December 22, 1897. | |
| Red Corpuscles | 3720000 |
| Hemoglobin, per cent. | 62 |
| III. EXAMINATION. Date, January 8, 1898. | |
| Red Corpuscles | 3916000 |
| Hemoglobin, per cent. | 62 |
| IV. EXAMINATION Date, January 16, 1898. | |
| Red Corpuscles | 3890000 |
| Hemoglobin, per cent. | 65 |
| DURATION OF TREATEENT | 51 days |
| GAIN (Absolute) — Red Corpuscles (in 1000's) | 440 |
| GAIN (Absolute) — Hemoglobin, per ct. | 5 |
| GAIN (Relative) — Red Corpuscles, per cent. | 13 |
| GAIN (Relative) — Hemoglobin, per ct. | 8 |

flow. Microscopic report showed a relative gain: red blood corpuscles, 13 per cent.; hemoglobin, 8 per cent.

CASE 7.—Mrs. J., aged 48 years, widow; mother of a large family. Cardinal feature of case was recurrent cephalalgia at intervals of several days. This case reported an improvement as to the intensity and duration of headaches, after the period of fourteen days of treatment. Only two blood examinations were made. A further opportunity to observe this patient did not present itself, in consequence of her falure to continue the treatment. Microscopic examination showed a relative gain: red blood corpuscles, 14 per cent.; hemoglobin, 13 per cent.

CASE VII.

| | | |
|---|---|---|
| I. EXAMINATION. (Beginning of Treatment.) | | |
| | Date, November 30, 1897. | |
| | Red Corpuscles | 3210000 |
| | Hemoglobin, per cent. | 60 |
| II. EXAMINATION. | | |
| | Date, December 14, 1897. | |
| | Red Corpuscles | 3670000 |
| | Hemoglobin, per cent. | 68 |
| DURATION OF TREATMENT | | 14 days |
| GAIN (Absolute) | Red Corpuscles (in 1000's) | 460 |
| | Hemoglobin, per ct. | 8 |
| GAIN (Relative) | Red Corpuscles, per cent | 14 |
| | Hemoglobin, per ct. | 13 |

CASE 8.—H. F., aged 18 years, school-teacher, unmarried. Symptomatology of neurasthenia. Malaria was a complicating feature. Amenorrhea for past six months was the principal symptom for which she consulted me. Aside from a course of quinine to eradicate the malarial feature, I exclusively gave "Pepto-Mangan." After forty-seven days' treatment she was apparently much improved, her menses having appeared in the interim. Microscopic examination showed a relative gain: red blood corpuscles, 9 per cent.; hemoglobin, 22 per cent.

CASE VIII.

| | | |
|---|---|---|
| I. EXAMINATION. (Beginning of Treatment.) | | |
| | Date, November 30, 1897. | |
| | Red Corpuscles | 2970000 |
| | Hemoglobin, per cent. | 42 |
| II. EXAMINATION. | | |
| | Date, January 8, 1898. | |
| | Red Corpuscles | 3100000 |
| | Hemoglobin, per cent. | 49 |
| III. EXAMINATION. | | |
| | Date, January 16, 1898. | |
| | Red Corpuscles | 3250000 |
| | Hemoglobin, per cent. | 51 |
| DURATION OF TREATMENT | | 47 days |
| GAIN (Absolute) | Red Corpuscles in 1000's) | 280 |
| | Hemoglobin, per ct. | 9 |
| GAIN (Relative) | Red Corpuscles, per cent | 9 |
| | Hemoglobin, per ct. | 22 |

CASE 9.—Mrs. L., aged 42 years, married, three children. Comes from neuropathic family, one uncle an epileptic. Has always been quite delicate and anemic. Since sudden death of husband has manifested great irritability of temper. Loses control of herself upon the slightest provocation. Cries easily, but not melancholic. Peculiarly apprehensive of sudden death; imagines upon retiring, that she will never awake; paroxysmal attacks of anxiety, and fatigued upon the slightest exertion. Anorexia. Habitual constipation. Sleeps restlessly. Patient although still very pale, after taking "Pepto-Mangan" for twenty-seven days, began to manifest a general improvement. Microscopic report showed a relative gain: red blood corpuscles, 11 per cent.; hemoglobin, 12 per cent.

CASE IX.

I. EXAMINATION.
(Beginning of Treatment.)
Date, January 2, 1898.
Red Corpuscles...... ...3720000
Hemoglobin, per cent.. 54

II. EXAMINATION.
Date, January 22, 1898.
Red Corpuscles.........4105000
Hemoglobin, per cent.. 60

III. EXAMINATION.
Date, January 29, 1898.
Red Corpuscles.... ...4130000
Hemoglobin, per cent.. 64

DURATION OF TREATMENT......27 days

GAIN (Absolute) { Red Corpuscles (in 1000's).......... 410; Hemoglobin, per ct.. 10 }

GAIN (Relative) { Red Corpuscles, per cent........... 11; Hemoglobin, per ct.. 12 }

CASE 10.—Mrs. P., aged 36 years, married, no children. Family history predisposed to tuberculosis. Physically in good health. Since cyclone, May 27, 1896, when her house was totally destroyed, and she narrowly escaped death, she developed nervous headaches; later on she manifested a listless and apathetic condition. Sleeps excellently, but does not feel refreshed upon awakening. Complains of drowsiness. Marked irritability of temper. Appetite fair, but nervous dyspepsia. Boards with sister as she cannot muster courage to manage a household of her own. After taking "Pepto-Mangan" for twenty-five days she began to feel much brighter and better, but still occasionally lapses into her former indifferent mood. Color better, and nervous dyspepsia greatly relieved. Microscopic report

CASE X.

I. EXAMINATION.
(Beginning of Treatment.)
Date, January 4, 1898.
Red Corpuscles.... ...3124000
Hemoglobin, per cent.. 56

II. EXAMINATION.
Date, January 14, 1898.
Red Corpuscles..........320000
Hemoglobin, per cent... 57

III. EXAMINATION.
Date, January 22, 1898.
Red Corpuscles.........3250000
Hemoglobin, per cent.. 62

IV. EXAMINATION.
Date, January 29, 1898.
Red Corpuscles.........3460000
Hemoglobin, per cent.. 68

DURATION OF TREATMENT......25 days

GAIN (Absolute) { Red Corpuscles (in 1000's) ......... 336; Hemoglobin, per ct.. 12 }

GAIN (Relative) { Red Corpuscles, per cent .............. 12; Hemoglobin, per ct. 12 }

showed a relative gain: red blood corpuscles, 12 per cent.; hemoglobin, 12 per cent.

CASE XI.

I. EXAMINATION.
(Beginning of Treatment.)
Date, January 5, 1898.
Red Corpuscles.........3856000
Hemoglobin, per cent.. 63

II. EXAMINATION.
Date, January 14, 1898.
Red Corpuscles.........4001000
Hemoglobin, per cent. 65

III. EXAMINATION.
Date, January 22, 1898.
Red Corpuscles.........4051000
Hemoglobin, per cent. 71

IV. EXAMINATION.
Date, January 29, 1898.
Red Corpuscles........4120000
Hemoglobin, per cent. 75

DURATION OF TREATMENT .... 24 days

| | | |
|---|---|---|
| GAIN (Absolute) | Red Corpuscles (in 1000's)............... | 264 |
| | Hemoglobin, per ct. | 12 |
| GAIN (Relative) | Red Corpuscles, per cent...... ....... | 11 |
| | Hemoglobin, per ct. | 12 |

CASE 11.—Mr. M., aged 29 years. Family history tuberculous. His avocation was that of a "book-maker" during the past few years. The strain of gambling and the consequent excitement and worry have made him a nervous wreck. Jerky and fidgety at all times. Inability to concentrate his mind any time. Suffers from nightmares and phantasmagoria during sleep, which is consequently much disturbed. Is troubled with constipation and greatly impaired digestion. Anorexia marked. Much reduced in weight. Although always fatigued and depressed, he constantly walks to relieve his pent-up nervous irritability. Dreads to be alone for fear something may happen to him. After the administration of "Pepto-Mangan" for twenty-four days, patient reports a general improvement, especially as to his appetite and the relief of his indigestion. Microscopic report showed a relative gain: red blood corpuscles, 11 per cent.; hemoglobin, 12 per cent.

CASE XII.

I. EXAMINATION.
(Beginning of Treatment.)
Date, January 16, 1898.
Red Corpuscles.... ....2985000
Hemoglobin, per cent...... 49

II. EXAMINATION.
Date, January 23, 1898.
Red Corpuscles.........3120000
Hemoglobin, per cent.. 53

DURATION OF TREATMENT ......7 days

| | | |
|---|---|---|
| GAIN (Absolute) | Red Corpuscles (in 1000's)............... | 135 |
| | Hemoglobin, per ct. | 4 |
| GAIN (Relative) | Red Corpuscles per cent................. | 5 |
| | Hemoglobin, per ct. | 8 |

CASE 12.—A. McG., aged 20 years, servant, unmarried. History showed the ordinary "symptom-group" of neurasthenia. After the short period of seven days, having taken but one bottle of "Pepto-Mangan," her condition was greatly alleviated. Microscopic report showed a relative gain: red blood corpuscles, 5 per cent.; hemoglobin, 8 per cent.
